# Barriers to and competency with the use of metered dose inhaler and its impact on disease control among adult asthmatic patients in Ethiopia

**DOI:** 10.1186/s12890-020-1081-6

**Published:** 2020-02-21

**Authors:** Tadesse Melaku Abegaz, Efrata Ashuro Shegena, Natnael Fentie Gessie, Eyob Alemayehu Gebreyohanns, Mohammed Assen Seid

**Affiliations:** 1 University of South Australia, School of Pharmacy and Medical Sciences, Adelaide, Australia; 2 University of Gondar, College of Medicine and Health Sciences, School of Pharmacy, Gondar, Ethiopia

**Keywords:** Asthma, Control, Barrier, Competency, Metered, Dose, Inhaler

## Abstract

**Background:**

Asthma is one of the chronic diseases which affects the airway, and inhalers are the preferred medications to treat this problem. Improper inhalational technique leads to decreased efficacy of the medication by reducing its deposition in the lungs. The aim of this study was to assess the barriers to and competency with the use of Metered Dose Inhaler (MDI) and its impact on disease control among adult asthmatic patients.

**Methods:**

A prospective cross-sectional study was conducted in University of Gondar comprehensive specialized hospital outpatient department (OPD) chronic follow up from 12-March-2018 to 15-May- 2018. Patients were interviewed face to face with questions which determined their competency, asthma control level and barriers for inhaler use.

**Result:**

Overall, 307 asthmatic patients were included in the study. More than half of participants were females, 170 (55.4%) and lived in urban area 185 (60.3%). The mean age of the respondents was 51.77 years with a standard deviation of ±15.40. The cost of medication, 282 (91.9%) and the perception that medication should be used in response to symptoms but not on a regular basis 277 (90.2%) were the most identified barriers. Only 56 (18.2%) were competent for Metered Dose Inhaler use (MDIU) and 17 (5.5%) patients had well controlled asthma. Being not competent AOR 0.168[0.41–0.687] was one of the factors decreasing asthma control.

**Conclusion:**

Generally from this study, cost of the medication and the perception that medication should be used only for symptoms were the major identified barriers that affect the MDI use among asthmatic patients. Patients show very poor competence to their MDI which in turn led to poor asthma control. So, patients need to be taught the correct inhaler technique in the hospital and pharmacy while they came for follow up every time.

## Background

Asthma is disease of the airway, which is one of the major chronic disorders affecting more than 300 million people worldwide and nearly 25 million people in the United States [1, 2]. It is characterized by variable and recurring symptoms, airflow obstruction, bronchial hyper responsiveness, and an underlying inflammation [1–3]. Even though it affects all people, the prevalence in developing countries is high and associated with increased treatment cost, and disease burden. Wheeze, shortness of breath, chest tightness and cough are some of the symptoms seen in asthmatic patients [1]. Nowadays, asthma has emerged as an important public health problem in many countries worldwide. The prevalence of asthma has increased more than 10-fold in many affluent societies since the 1960s [4]. A self-reported study done in 70 countries showed that around 623 million people are having asthmatic symptom [3]. A systematic analysis which estimates the prevalence of asthma in Africa suggested that there is increment in asthma prevalence in Africa over the past two decades in which 74 million(11.7%) population were affected in 1990 but 120 million(12.8%) in 2010 [5] and even though, the prevalence of asthma in Africa countries is moderate, its impact is high [6]. Also, Ethiopia, according to the latest world health organization (WHO) data published in 2014, deaths caused by asthma reached 1.12% of total deaths and ranked 18th from the world [7].

Despite the availability of effective therapy, asthma control didn’t meet the specified goal in many countries. A cross sectional study done in 29 countries in North America, Europe, Japan, and the Asia-Pacific region shows asthma control is suboptimal [4]. Asthma was also poorly controlled in a high proportion of respondents in Asia, in which more than half of the patients’ experience day time wheeze, cough and night time awakening and most of the patients have poor understanding about ICS [8]. There is a big difference seen between the treatment goals and the current level of asthma control achieved in the general population in which epidemiologic data also suggests that many subjects with asthma have poorly controlled disease [9]. The reason behind this could be suboptimal or non-adherence to inhaled therapies for asthma and COPD which is associated with poor symptom control, higher healthcare utilization and healthcare costs, and reductions in health-related quality of life [10].

A meta-analysis which systematically reviews the errors in inhaler use from 1975 to 2014, concludes incorrect inhaler use in patients with asthma and COPD is unacceptably high [11]. This may be a major obstacle for achieving good asthma control which is pointing to an urgent need for new approaches to education and drug delivery. Incorrect use of inhalers can lead to detrimental effects on asthma management [12] and worsens the clinical outcome [13].

Inhaler mishandling remains a serious issue and patients need education on the inhaler use by health caregivers [13]. Patient’s poor knowledge regarding their disease [14, 15] and poor competency among dispensers [16–18] are among factors leading to improper use.

In Ethiopia, there are different types of inhalational asthmatic medications including, salbutamol, beclomethasone, budesonide, fluticasone, salmeterol and formoterol. MDIs are the most available and used devices for asthma treatment in Ethiopia. Yet, the dry powder is in the drug formulary, but not available in pharmacies [19]. However, there is paucity of data regarding the level of competency for inhaler use in asthmatic population. Strategies to scale-up the elimination of modifiable barriers to inhaler use have not also been exhaustively implemented. Investigation of the potential barriers to the use of asthma inhalers, determination of competency of patients for MDI and asthma control would enable for decreasing the potentially modifiable barriers which leads to a better disease control, minimize cost and further complications among asthmatic patients. Therefore, this study aimed to assess the barriers to and competency with the use of metered dose inhaler and its impact on disease control among adult asthmatic patients.

## Methods

### Study setting and period

This study was conducted in University of Gondar comprehensive specialized hospital outpatient department (OPD) chronic follow up from 12-March-2018 to 15-May- 2018. UoGCSH is found in Gondar town North West Ethiopia, which is 727 km far from the capital Addis Ababa. The hospital gives services for 5 million people and has both inpatient and outpatient departments. The chronic OPD gives service for chronic patients and asthma is one of the services given in the chronic OPD.

### Study design

A cross sectional prospective study was conducted on asthmatic patients.

### Population

#### Source population

All patients who were diagnosed for asthma and who came for follow up to UoGCSH during the study period were our source population.

#### Study population

All adult asthmatic patients who were using at least one metered dose inhaler asthma medication and who came for outpatient visit were our study population.

### Inclusion and exclusion criteria

All adult asthmatic patients using inhaler who came for follow up during the study period were included and patient’s age below 18 and who had communication problem were excluded.

### Sample size determination and sampling technique

According to recent data from the record of the OPD of UoGCSH an estimated number of 1525 of asthma patients were supposed to attend the hospital. So far, the proportion of asthma population who had controlled asthma and level of competency was not known in north Ethiopia. Therefore, the final sample size was obtained using single population proportion and reduction formula. According to single population proportion formula the estimated sample size was 384. But the total asthmatic population attending the hospital was 1525 which was below 10,000, hence we applied a reduction formula to obtain a final sample size of 307 patients. We described the steps we followed to calculate the final sample size as follows.

$$ {\displaystyle \begin{array}{l}\mathrm{n}={\mathrm{z}}^2\mathrm{p}\;\left(1-\mathrm{p}\right)/{\mathrm{w}}^2\\ {}\mathrm{n}=\mathrm{sample}\kern0.17em \mathrm{size}\kern2.759996em \mathrm{p}=\mathrm{population}\ \mathrm{proportion},50\%(0.5)\\ {}\mathrm{w}=\mathrm{margin}\ \mathrm{of}\ \mathrm{error}\ \left(5\%\right)=0.05\kern1.56em \mathrm{z}=1.96\ \left(\mathrm{for}\ 95\%\mathrm{confidence}\ \mathrm{interval}\right)\\ {}\mathrm{n}=\frac{(1.96)^2\ast 0.5\ast 0.5}{(0.05)^2}=384.16\end{array}} $$

Since the total asthma population in the study setting is less than 10,000, a minimum sample size required. Hence, we used the following reduction formula to reach a final sample size.

$$ \mathrm{N}=\mathrm{number}\ \mathrm{of}\ \mathrm{asthmatic}\ \mathrm{patients}\ \mathrm{attending}\ \mathrm{UoGCSH}=1525 $$

$$ \mathrm{S}\left(\mathrm{final}\ \mathrm{sample}\ \mathrm{size}\right)=\mathrm{n}/\left(1+\mathrm{n}/\mathrm{N}\right)=384.16/\left(1+384.16/1525\right)=306.9\approx 307. $$

### Study variable

#### Dependent variables

Level of asthma control, competency to MDI and barriers to the use of MDI were the dependent variables of this study.

#### Independent variables

The predictive value of the study were socio demographic characteristics of the patients including age, gender, residence, educational status, comorbidity and number of type of medication, disease length and follow up.

### Data quality control technique

Data collectors were having knowledge on contents of questionnaire, data collection methods and ethical concerns before starting the data collection. The questionnaire was pre-tested on (5%) asthmatic patients attending their follow up at UoGCSH that was used prior to the actual data collection to modify the questionnaire. The content of the questionnaire was translated in to Amharic in order to maintain unbiased response. The filled questionnaire was checked daily for completeness by the principal investigators. The internal validity of the questionnaire was examined and demonstrated a Cronbach alpha value of more than 0.78. A specific number was given for each patient once they are done with the interview in order to avoid repetition for the next time.

### Data collection method and data collection procedure

Data was collected by two undergraduate clinical pharmacy students. A structured questionnaire was prepared from previous literatures to assess barriers to MDI use. The questionnaire contains 25 barrier questions in five different domains [20, 21]. Socio demographic and clinical data of patients were incorporated in our questionnaire. A 5-item asthma control test questions were used to evaluate the level of control of asthma [22]. Competency was scored using the National Asthma Education and Prevention Programs of America (NAEPP) step criteria for demonstration of an MDI [23]. Information on socio demographic, past and current medication use was taken from patients’ card and patients were interviewed the rest of the questioner for detailed information. Patients were asked to show how they use their inhaler to assess their competency to the MDI. However, we did not use another adherence measurement tool to assess the overall medication adherence.

### Data analysis

All the statistical analysis was carried out using SPSS, version 20. Descriptive statistics was presented using means with standard deviation (±SDs) and percentages (%). Graphs and tables were utilized to represent the data. A 95% confidence interval with *P* value of < 0.05 was considered significant. Binary logistic regression was employed to determine factors associated with asthma control and MDI use.

### Operational definition

Not well controlled; according to the asthma control test if the overall score of the test is less than or 19 the asthma symptoms are very poorly controlled.

Well controlled: if the score is above 20, the asthma symptoms are considered well controlled.

Competent - According to the NAEPP evaluation tool, patients who scored 7 or above and demonstrate the essential steps correctly were classified as competent to the MDI.

Poor competency - patients who didn’t perform all the essential steps and scored below 7 were considered as having poor competency or incompetent.

## Results

### Socio demographic characteristics of respondents

Overall, 307 asthmatic patients were included in the study. The mean age of the respondents was 51.77 with a standard deviation of ±15.40. More than half of participants were females, 170 (55.4%) and lived in urban area 185 (60.3%). More than one-half of the patients 175 (57.0%) didn’t get formal education and one third of the patients, 103 (33.6%) had no occupation. The detailed socio demographics of the patients are presented in Table 1.

**Table 1:** The socio demographic characteristics of asthma patients attending UoGCSH in 2017/18 (*n*=307)

Characteristics	N(%)
Age (mean ± SD)	51.77 ± 15.40
Sex	
Female	170 (55.4%)
Male	137 (44.6%)
Educational status	
No formal education	175 (57.0%)
Primary school	31 (10.1%)
Secondary school	50 (16.3%)
University	51 (16.6%)
Occupation	
Government bureau	45 (14.7%)
Private company/business	102 (33.2%)
Farmer	46 (15.0%)
Student	11 (3.6%)
None	103 (33.6%)
Residence	
Urban	185 (60.3%)
Rural	122 (39.7%)

### Clinical characteristics of patients

The mean duration of the disease in years was 11.22 with a standard deviation of ±9.92. All of the respondents were diagnosed with bronchial asthma. During exacerbation, nearly half of the patients showed symptoms continuously or all over the day, 149 (48.5%). Majority of the patients had regular follow up to the chronic OPD, 293 (95.4%) and more than half of them had comorbid disease, 164 (53.4%). The average period between visits was 1.31 ± 0.63 months. More than half of the patients 191 (62.2%) use beclomethasone with salbutamol for the treatment of Asthma (Table 2).

**Table 2:** The clinical characteristics of asthma patients attending UoGCSH in 2017/18 (*n* = 307)

Clinical characteristics	N (%)
Duration of the disease in year (mean ± SD)	11.22 ± 9.92
Type of Asthma	
Bronchial asthma	307 (100%)
frequency of symptoms during exacerbation	
1–2 times	46 (15%)
3–5 times	58 (18.9%)
5–10 times	54 (17.6%)
Continuously (all over the day)	149 (48.5%)
Family history	
Yes	92 (30%)
No	215 (70%)
regular follow up	
Yes	293 (95.4%)
No	14 (4.6%)
Duration of appointment in month (mean ± SD)	1.31 ± 0.63
Comorbidity	
Yes	164 (53.4%)
No	143 (46.6%)
Type of complication	
Heart failure	34 (11.1%)
Hypertension	67 (21.8%)
COPD	18 (5.9%)
Other	45 (14.7%)
No complication	143 (46.6%)
Name of the medication	
Salbutamol	116 (37.8%)
Salbutamol with Beclomethasone	191 (62.2%)

### Barriers to MDI use

**Fig. 1:**
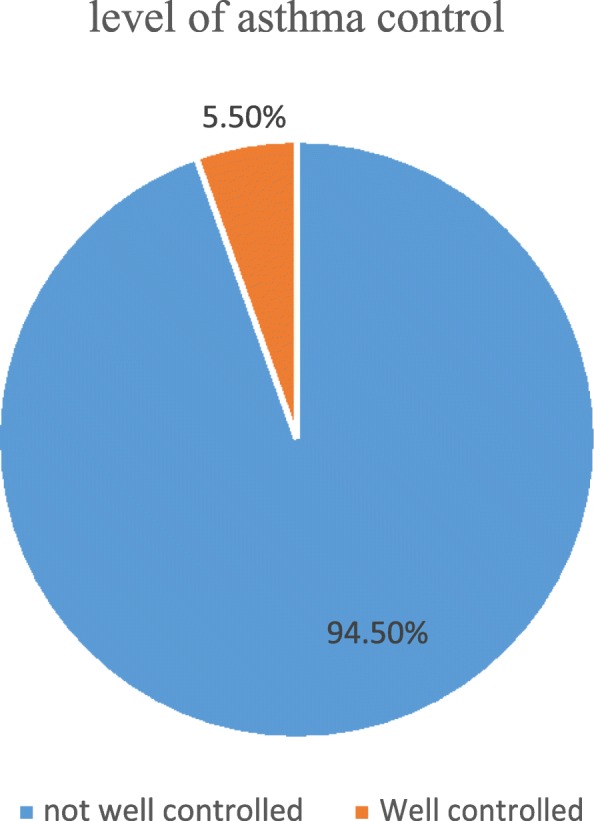
Top ten barriers encountered by asthma patients attending UoGCSH in 2017/18

Different barriers were delineated in five major domains in the table below. Among the cognitive barriers (CBs), “the perception that medication should be used in response to symptoms only 277 (90.2%)”, “the belief of decreasing effectiveness of the medication over time 270 (87.9%)”, and “fears of addiction to their medication 266 (86.6%)” were the three most common barriers, respectively. Motivation and preferences related barriers (MPRBs) including “preference for other non-pharmacological approach 212 (69.1%)” and “preference for restriction of daily physical activity instead of taking medication 196 (63.8%) were among the major barriers. Whereas, failing to brush teeth 236 (76.9%) and cost of the medications 282 (91.9%) were among the common practical implementation barriers (PIBs). About family and physician related barriers (FPRBs), “centering the asthma management on prescribed medication 274 (89.3%)” was a frequently stated barrier. Nearly ninety percent 274 (89.3%) of patients reported that contradictory messages provided by different health care professionals also affect their inhaler use among the health care related barriers (HCRBs) (Table 3). The frequencies of top ten barriers to MDI are indicated below in Fig. 1.

**Table 3:** Barriers to the use of MDI among asthma patients attending UoGCSH in 2017/18 (*n*=307)

Barriers to MDI use		Yes N(%)	No N(%)
Cognitive barriers (CBs)			
B1	The belief of the patient that their asthma is not serious to take the medication	117 (38.1%)	190 (61.9%)
B2	Fears of addiction or dependence to their medication	266 (86.6%)	41 (13.4%)
B3	The perception that medication should be used in response to symptoms, and not on a regular basis	277 (90.2%)	30 (9.8%)
B4	The belief of decreasing effectiveness of the medication over time	270 (87.9%)	37 (12.1%)
B5	Patients had inadequate or limited knowledge about their medication	264 (86%)	43 (14%)
B6	The fear of adverse effects of medication associated with use of an inhaler	262 (85.3%)	45 (14.7%)
B7	Inadequate or limited knowledge whether they were taking the right medication	189 (61.6%)	118 (38.4%)
B8	Inadequate or limited knowledge whether they were using the appropriate technique	195 (63.5%)	112 (36.5%)
B9	Inadequate or limited knowledge whether the medication they were taking was compatible with medications taken for other conditions	248 (80.8%)	59 (19.2%)
Motivation and preferences related barriers (MPRBs)			
B10	Forgetfulness	125 (40.7%)	182 (59.3%)
B11	Lack of motivation	132 (43%)	175 (57%)
B12	Preference for other non-pharmacological approach	212 (69.1%)	95 (30.9%)
B13	Preference for restriction of daily physical activity instead of taking medication	196 (63.8%)	111 (36.2%)
Practical implementation (PIBs)			
B14	Having to brush their teeth after its use	71 (23.1%)	236 (76.9%)
B15	Uncomfortable for the use of the inhaler	138 (45%)	169 (55%)
B16	Having trouble to take the medication more than once a Day	143 (46.6%)	164 (53.4%)
B17	The cost of medication	282 (91.9%)	25 (8.1%)
Family and physician related barriers (FPRBs)			
B18	Disagreements between parents	67 (21.8%)	240 (78.2%)
B19	Unclear language and instruction	229 (74.6%)	78 (25.4%)
B20	Uninformed about the disease or diagnosis	253 (82.4%)	54 (17.6%)
B21	The severity of the disease remained unclear to them	245 (79.8%)	62 (20.2%)
B22	Physicians centered the asthma management strategy solely on prescribed medications	274 (89.3%)	33,910.7%)
Health care related barriers (HCRBs)			
B23	The lack of a structured follow-up plan	261 (85%)	46 (15%)
B24	Lack of specialists treating asthma	262 (85.3%)	45 (14.7%)
B25	Contradictory messages provided by different health care professionals	274 (89.3%)	33 (10.7%)

*B* Barrier

#### Competency with MDI

All the patients were asked to show how they use their inhaler and almost all patients perform correctly the second and the last step which is to remove the cap, 304 (99%) and to replace the mouth piece cover, 300 (97.7%) respectively. The most missed or incorrectly performed step was to breath out slowly, 250 (81.4%). Nearly fifty percent of the respondents didn’t hold the inhaler in upright position, 153 (49.8%). The study shows that approximately three-fourth 230(74.9 %) of the patients did not hold their breath for 10-20 seconds after taking the drug, and they did not exhale and wait one minute before the second dose (Table 4). This study revealed that only 56 (18.2%) patients scored above 7 but all of them did not perform all the essential steps correctly, according to the NAEPP.

**Table 4:** Competency with MDI among asthma patients attending UoGCSH in 2017/18 (*n* = 307)

Steps	Competency	Correct N (%)	Incorrect or missed N(%)
1*	Shake the contents well	273 (88.9%)	34 (11.1%)
2*	Remove the cap	304 (99%)	3 (1%)
3	Hold the inhaler upright	154 (50.2%)	153 (49.8%)
4	Tilt the head back slightly	65 (21.2%)	242 (78.8%)
5*	Breath out slowly	57 (18.6%)	250 (81.4%)
6*	Hold the inhaler in the mouth with the lips tightly sealed around.	241 (78.5%)	66 (21.5%)
7*	Begin breath in slowly and deeply through the mouth and actuate the canister once	133 (43.3%)	174 (56.7%)
8*	Hold breath for 10–20s	77 (25.1%)	230 (74.9%)
9	Exhale and wait one minute before the second dose	78 (25.4%)	229 (74.6%)
10	Shake again before the second dose	222 (72.3%)	85 (27.7%)
11	After use, replace the mouth piece cover	300 (97.7%)	7 (2.3%)
Level of competency		Competent	Not competent
		56 (18.2%)	251 (81.8%)

*essential steps according to NAEPP

**Fig. 2:**
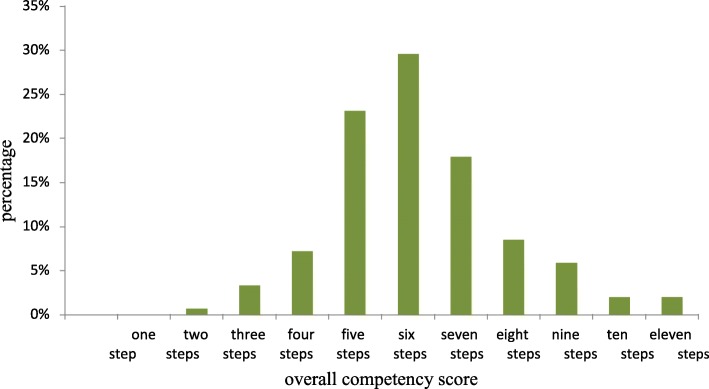
Overall score of the respondents on MDI technique (%) among asthmatic patients attending UoGCSH in 2017/18 (*n* = 307)

From overall score of the respondents, majority of patients perform six steps correctly, 29.6% followed by five steps, 23.1% while few patients demonstrate for two steps, 0.7% and 10-11 steps, 2% (Fig. 2).

#### Level of asthma control

**Fig. 3:**
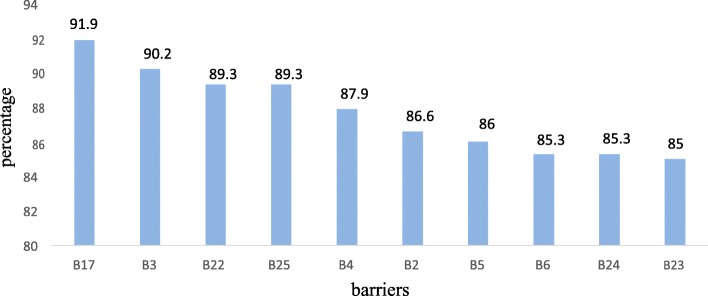
Level of asthma control among asthmatic patients in UoGCSH in 2017/18

Using asthma control test, the overall asthma control was classified as” not well controlled”. Accordingly, it was found that in almost ninety five percent, 290 (94.5%) of patients, Asthma was not well controlled while only 17 (5.5%) patients had well controlled asthma (Fig. 3).

#### Factors affecting MDI competency

Binary logistic regression was undertaken to find out the factors affecting the MDI competency. Among the factors associated with MDI competency, length of the disease was significantly affecting competency to MDI. As the patient had long duration of disease the competency rate slightly increased: COR 1.029[1.002-1.057], AOR 1.040[1.005-1.075] with *p* value of <0.05. However, other variables did not show any correlation with MDI competency on binary logistic regression (Table 5).

**Table 5:** Factors affecting MDI competency among asthmatic patients in UoGCSH in 2017/18

Variables	Competent	Incompetent	COR 95%CI	AOD 95%CI
	56 (18.2%)	251 (81.8%)		
Age	51.71 ± 16.38	51.78 ± 15.21	1.000[0.981–1.019]	1.004 [0.976–1.034]
Sex				
Males	25(8.14%)	112(36.48%)	1.001[0.559–1.792]	1.085 [0.532–2.211]
Female	31(10.09%)	139(45.27%)	1	1
Education				
No formal education	28(9.1%)	147(47.88%)	1	1
Primary school	8(2.6%)	23(7.49%)	0.889[0.389–2.030]	0.771 [0.238–2.502]
Secondary school	11(3.5%)	39(12.70%)	1.623[0.551–4.778]	1.210 [0.341–4.295]
University	9(2.93%)	42(13.68%)	1.316[0.493–3.517]	0.999 [0.333–2.993]
Occupation				
Government bureau	7(2.2%)	38(12.37%)	1	1
Private company/business	23(7.49%)	79(25.73%)	0.814[0.316–2.101]	0.474 [0.210–2.664]
Farmer	4(1.3%)	42(13.69%)	1.287[0.652–2.543]	1.579 [0.681–3.662]
Student	3(0.97)	8(2.6%)	0.421[0.135–1.317]	0.506[0.135–1.892]
None	19(6.18%)	84(27.36%)	1.658[0.402–6.840]	2.040[0.340–12.247]
Residence				
Urban	36(11.7%)	149(48.5%)	1.232[0.675–2.249]	0.804 [0.392–1.650]
Rural	20(6.5%)	102(33.22%)		1
Duration of the disease in year (mean)	13.81 ± 12.31	10.65 ± 9.25	1.029[1.002–1.057]*	1.040[1.005–1.075]*
No of exacerbation per day				
1–2 times	6(1.95%)	40(13.02%)	1	1
3–5 times	14(4.56%)	44(14.3%)	0.648[0.250–1.678]	0.598[0.215–1.663]
5–10 times	8(2.6%)	46(14.98%)	1.375[0.664–2.849]	1.586[0.725–3.465]
Continuously	28(9.1%)	121(39.4%)	0.752[0.319–1.769]	0.755[0.302–1.892]
Family history				
Yes	20(6.51%)	72(23.5%)	1.381[0.75–2.545]	1.330[0.691–2.561]
No	36(11.72%)	179(58.3%)	1	1
Any regular source of care (follow up)				
Yes	13(4.23%)	238(77.52%)	0.294[0.43–2.599]	3.095[0.334–28.662]
No	1(0.33%)	55(17.9%)	1	1
Duration of appointment in month (mean)	1.38 ± 0.65	1.29 ± 0.63	1.212[0.779–1.887]	1.294[0.767–2.183]
Comorbidity				
Yes	27(8.79%)	137(44.62%)	0.775[0.434–1.384]	0.651[0.335–1.264]
No	29(9.44%)	114(37.12%)	1	1
Name of the medication				
Salbutamol only	22(7.16%)	94(30.6%)	1	1
Salbutamol with Beclomethasone	34(11.07%)	157(51.14%)	1.081[0.597–1.958]	1.105[0.565–2.160]

**Table 6:** Factors affecting asthma control among asthmatic patients in UoGCSH in 2017/18

Variables	Controlled (17)	Uncontrolled (290)	COR 95%CI	AOR 95%CI
Age	55.29 ± 15.41	51.5 ± 15.23	1.017[0.983–1.051]	1.097 [0.21–1.178]
Sex				
Males	6(1.95%)	131(42.67%)	0.662[0.238–1.838]	1.47 [1.29–1.737]*
Female	11(3.58%)	159(51.79%)	1	1
Education				
No formal education	6(1.95%)	169(55.04%)	1	1
Primary school	5(1.62%)	26(8.46%)	0.568[0.137–2.356]	0.528 [0.43–6.540]
Secondary school	3(0.97%)	47(15.31%)	3.077[0.681–13.912]	3.468[0.307–39.231]
University	3(0.97%)	48(15.63%)	1.021[0.196–5.318]	0.877[.95–8.098]
Occupation				
Government bureau	4(1.30%)	41(13.35%)	1	1
Private company/business	8(2.60%)	94(30.61%)	3.252[0.697–15.176]	3.440[0.221–53.614]
Farmer	2(0.65%)	44(14.33%)	2.837[0.731–11.014]	2.330[0.312–17.431]
Student	0(0%)	11(3.58%)	1.515[0.245–9.389]	8.953[0.526–152.233]
None	3(0.97%)	100(32.57%)	.000[0.000	0.000[0.000
Residence				
Urban	14(4.56%)	171(55.7%)	0.308[0.87–1.095]	8.970[1.174–68.538]*
Rural	3(0.97%)	119(38.76%)	1	1
Duration of the disease (mean)	15.55 ± 13.85	10.96 ± 9.62	1.039[0.997–1.082]	1.035[0.974–1.101]
No of exacerbation per day				
1–2 times	5(1.62%)	41(13.35%)	1	1
3–5 times	2(0.651%)	56(18.24%)	2.474[0.746–8.206]	4.558[0.900–23.086]
5–10 times	3(0.97%)	51(16.6%)	0.724[0.146–3.594]	0.501[0.63–3.959]
Continuously	7(2.28%)	14,246.25%)	1.193[0.297–4.790]	1.268[0.161–9.987]
Family history				
Yes	6(1.95%)	86(28.01%)	1.294[0.464–3.610]	1.439[0.377–5.496]
No	11(3.58%)	204(66.45%)	1	1
Regular follow-up				
Yes	16(5.21%)	277(90.22%)	1	1
No	1(0.325%)	13(4.23%)	0.751[0.92–6.104]	0.045[0.002–0.931] *
Comorbidity				
Yes	8(2.6%)	156(50.81%)	0.764[0.287–2.034]	0.470[0.117–1.895]
No	9(2.93%)	134(43.65%)	1	1
Medication				
Salbutamol	12(3.9%)	104(33.87%)	1	1
Salbutamol with Beclomethasone	5(1.62%)	186(60.58%)	4.292[1.472–12.520]**	10.275[2.228–47.380]**
Level of competency				
Competent	7(2.28%)	49(15.96%)	1	1
Not competent	10(3.25)	241(78.5%)	0.290[0.105–0.800]*	0.168[0.41–0.687]*

*significant at *p* < 0.05 level, ** significant at *p* < 0.01 level

### Determinants of asthma control

Asthma control was affected by different independent variables. Accordingly, the binary logistic regression showed that asthma control was nearly 1.5 times better among male patients than females AOD 1.47 [1.29 – 1.737]. Patients living in urban area had almost nine times increase in their asthma control: AOR 8.970[1.174-68.538]. Patient who had no regular follow-up showed a decreased asthma control than their counter parts AOR 0.045[0.002-0.931]. Patients who were using both Beclomethasone and Salbutamol for the treatment of asthma had ten times control over the symptoms of asthma: COR 4.292[1.472-12.520], AOD 10.275[2.228-47.380]. As patients were not well competent to their inhaler, the level of asthma control decreased by nearly 83%; COR 0.290[0.105-0.800], AOR 0.168[0.41-0.687]. Other variables were not significantly correlated with level of asthma control (Table 6).

## Discussion

MDIs are the main stay therapy for the treatment of asthma as they directly propel the medication to the air ways [1, 2] which maximizes the efficacy of the medication and decreases the adverse effect [24]. Poor competency with the inhaler would lead to decreased amount of drug to reach to the airways than the needed amount [25]. The inadequate expertise of MDI use and the constellation of barriers adversely affect asthma control. Identification of the major barriers would help to design an intervention to avoid these obstacles [26]. Therefore, this study aimed to explore the level of competency with and barriers to, the use of MDI and to determine its impact on asthma control among patients attending the outpatient clinic of comprehensive specialized hospital in northwest Ethiopia.

The current study identified a total of twenty-five major barriers of MDIU in five main domains. Amongst the CBs, more than 90 % of the patients had the perception that medication should be used in response to symptoms only. Patients did not get the idea of taking long term treatment in the absence of symptoms; as a result, they will only use their anti-asthmatic medications for controlling symptoms. Some individuals could also anticipate that a regular administration of inhaled corticosteroids might lead to long term adverse effects which in turn play as a significant barrier for MDIU [27]. Our study also revealed that fear of adverse effect was seen in significant proportion of (85%) of asthmatic patients. A cohort study showed that level of medication-related attitudes and frequency of ADR were listed as modifiable barriers [28]. Another study finding suggests that, asthmatic patients should use their medication regularly to prevent the occurrence of symptomatology and exacerbation of asthma even in the absence of symptoms [29].

The cost of the medication was also stated as a major barrier in more than 90% of patients from the PIBs. A systematic review study done in 2009 also revealed that cost of medication is one of the direct medical costs affecting medication use among asthmatic patients [30]. This scenario is also seen in many chronically ill patients like asthma, heart failure, and depression, for which patients didn’t take their medication fully due to cost issue especially on those having low income. A retrospective cohort observational study done on diabetes, hypercholesterolemia, and hypertension patients also state that cost highly affects medication adherence [31]. This will lead to inadequate adherence and can quickly pose serious health problems [32, 33]. the problem is particularly important in the case of low-income countries including Ethiopia where expenditure on pharmaceuticals exceeds 39%. Beclomethasone and salbutamol were available in private pharmacies instead of public hospitals which affects the affordability of the medicines [34].

Further, lack of instruction given to patients about the use of MDI was stated as a major barrier in asthmatic patients in previous study [15] and in almost three-fourth of patients, lack of appropriate instruction was reported as a predominant barrier in our study. In addition to inadequate intricate of instructions, poor understanding of the instructions among asthmatic patients have been observed as an unintentional barrier to a treatment [35, 36]. Unclear knowledge and incorrect assumptions about medication and illness indicates the improper use of MDI [10]. Thus, to avoid biased information, patients require both verbal and practical instructions from skilled professionals. The instruction should also be explicit and correct information should be presented in transparent manner [25, 37]. The delivery of half wrong information for patients via inept health professionals leads to all wrong information for patients, which on the other hand results in lack of knowledge on the inhalation technique and subsequent reduction of therapeutic benefit [38]. Therefore, it is apparent that not only asthmatic patients but also health professional should demonstrate an adequate experience prior to tempting medication related counseling for patients.

In the present study, competency level with the inhaler was also determined using the NAEPP tool for the appropriate technique of inhaler use. It was found that few patients (18.2%) were able to perform more than seven steps out of eleven basic techniques of inhaler use. Among the essential steps according to NAEPP, the three most missed steps in our study were “breathe out slowly”, “hold breath for 10–20sec” and “begin breath in slowly and deeply through the mouth and actuate the canister once” respectively while removing the cap (88.7%) was the most correctly done procedure. This result is consistent with a study done to assess the inhalational technique among chronic respiratory patients which indicates about 60.3% patients failed to exhale before placing inhaler in the mouth, that makes it the most common obscuring error [25]. This is also observed in a study conducted at the University of Texas where 66% of patients failed to exhale before placing the inhaler [39]. Also, a systematic review of inhaler use error stated that the most frequent MDI errors were related to incoordination in breathing including, speed and/or depth of inspiration and inability to hold breath post inhalation [11]. From the findings of the present study, it can be highlighted that, emphasis should be given to these aspects while counseling our patients about the utilization of MDIs since the amount of dose instituted to the patient’s lung is determined based on the patients’ alacrity to apply the instrument on mouth along with canonical breathing [40]. Ironically, very poor MDI technique was seen among community pharmacists in Ethiopia, in many aspects of the steps of MDI use. Specifically, pharmacists were unable to demonstrate enough skills in breathing related techniques that are almost disregarded by the patients as well according to our study findings. Hence, breathing related tips are the potential area of improvement both at professional and patient level [16–18].

Furthermore, this study revealed that asthma control was very poor among patients using inhalers. Only 5.5% of patients had well controlled asthma as per the asthma control test results. Using the asthma control test P. Demoly et al., and his colleagues also discovered that uncontrolled asthma was nearly 50 % [41]. The difference with these results could be the study area difference between the European and developing countries in which level of literacy and economic advancement facilitates asthma control in Europe [33]. Study reports in Nigeria and Cameroon also stated that asthma control was found to be unacceptably poor [42, 43]. A cross-sectional study done in Jimma using the same tool showed that almost three fourth (76.1%) of the patients had uncontrolled asthma [44]. Significantly low level of asthma control revealed in our study awakens our mind to be more solicitous about the modifiable factors and to enervate their untoward impact on asthma control.

Moreover, this study indicated the associated factors affecting MDI competency using binomial logistic regression method. Accordingly, the duration of the disease significantly affected the competency of the patients for MDI. As the patients stayed with the disease for a long period of time than those with short duration of disease, their competence slightly increased. This was since, as the patients use the inhaler for longer period, they will have more experience plus in every follow up they have gotten enough instruction about the inhaler technique. Repeating the instruction in each follow up increases the ability to correctly perform the MDI [37, 45].

The current study indicated that asthma control was significantly affected with the patient’s competency to the inhaler. Patients who were not competent to their inhalers had decreased asthma control level by 83% than the competent ones. Poor asthma control correlation with the inhaler use could be explained by inadequate amount of medication reached to the lung. This result has been similar with other studies, in which a cross sectional study in chest clinic also concluded that poor competence to MDI among asthmatic patients would lead to poor asthma control [25]. Another study also explained that improper use of MDI which is mainly due to poor coordination is frequent and associated with poorer asthma control [46].

In addition, the present study indicated that patients living in urban area had adequate asthma control. In Ethiopia, most of the population living in the rural area did not get formal education. Due to that, they do have poor ability to read, understand medication instruction and to act on health information which will lead to poor competency to their MDI and asthma control. On the other hand, patients who can read and understand drug labels were found to be more likely to have good competence and asthma control [47, 48].

Further, females show inadequate asthma control as compared to males in our study. This result is also supported by other findings, a study done in Cameroon finds that women were more likely to have inadequate asthma control [43]. It is also stated females were more likely to be diagnosed and suffer from asthma in which the physiologic mechanism for these differences are not well understood [49]. And in our country Ethiopia, most of the females spent their time doing house hold activities including smoking from wood or kerosene that may exacerbate the asthmatic attack. It is well noticed that smokes and polluted air are associated with environmental exposures which will exacerbate asthma [1].

Our study has also reported that, patients who were prescribed for both beclomethasone and salbutamol had better asthma control. In contrary, significant number of patients (33.87%) on salbutamol monotherapy failed to achieve good asthma control. Initiation of maintenance therapy with ICS combined with β 2 agonists had greater improvements in pulmonary function and symptom control [50]. ICSs are most potent and effective anti-inflammatory medication used for long term asthma control while β 2 agonists are used only for acute symptoms and exacerbation as relivers [1]. Using combination of β 2-agonists and corticosteroid inhaler therapy are therefore a logical advance and results in effective control of asthma in most patients than having a mono therapy [51]. From our finding, patients with regular follow up had controlled asthma. This could be since, when patients had a regular follow-up the level of their competency to use the inhalers increase and we noticed from our study that competency improves the diseases control [52].

### Strength and limitation of the study

In general, this study provided detailed information on factors affecting MDIU, competency to MDI and asthma control. The prospective nature of the study enabled us to gather complete information since data was collected directly from the patients. In addition, there were no adequate literatures done in this research area. Hence, the present study will help to fill the unprecedented evidence gap in our set-up and to come up with solution for the problems in the future perspective. Also, while collecting the data, the patients were taught the correct inhaler technique, about major barriers and the main exacerbating factors of the disease.

Despite the strength of our study, it is realized that our study had several limitations. For one, the study is done in a single population which is mono-centered, and it will make the study difficult to generalize the findings for large population. In addition, some factors for instance, the lifestyle of individual patients, that could affect asthma control were not exhaustively explored in the current study. Further, the method we applied to evaluate the MDI use is not standardized.

## Conclusion

To conclude, this study showed that different barriers affect for MDI use among asthma patients. Of these, cost of the medication and the perception that medication should be used only for symptoms are the major barriers. Our study indicated that patients showed very poor competency to their MDI. It was found that only 18% of patients were competent and almost 95% of the patients had poor asthma control. Many different independent variables have been identified as determinant for the level of asthma control in our study. Among the factors, being male, urban residence, duration of appointment, type of medication and level of competency significantly affected the asthma control. Based on our finding we recommend UoGCSH health care administrates to give different short trainings on MDI appropriate technique for the physicians, pharmacists and all other health care workers so that they can teach the patients to properly administer their medications.

## Supplementary information

**Additional file 1.** Barrier and competency assessment questionnaire (BCA-Q).

## Data Availability

The data that support the findings of this study are available on request from the corresponding author, [TMA].

## Abbreviations

ACT: Asthma Control Test
CBs: Cognitive Barriers
COPD: Chronic Obstructive Pulmonary Disease
DPI: Dry powder inhaler
FPRBs: Family and physician related barriers
GINA: Global initiative for asthma
HCRBs: Health care related barriers
ICS: Inhaled Corticosteroids
MDI: Metered Dose Inhaler
MDIU: Metered dose inhaler use
MPRBs: Motivation and preferences related barriers
NAEPP: National Asthma Education and Prevention Programs of America
NWC: Not well controlled
OPD: Out Patient Department
PIBs: Practical implementation
RWS: Rate of wrong steps
SPSS: Statistical Package for Social Sciences
UoGCSH: University of Gondar comprehensive specialized hospital
WHO: World Health Organization
