# Correction to: Barriers to and competency with the use of metered dose inhaler and its impact on disease control among adult asthmatic patients in Ethiopia

**DOI:** 10.1186/s12890-020-1122-1

**Published:** 2020-04-20

**Authors:** Tadesse Melaku Abegaz, Efrata Ashuro Shegena, Natnael Fentie Gessie, Eyob Alemayehu Gebreyohannes, Mohammed Assen Seid

**Affiliations:** 1 University of South Australia, School of Pharmacy and Medical Sciences, Adelaide, Australia; 2 University of Gondar, College of Medicine and Health Sciences, School of Pharmacy, Gondar, Ethiopia

**Correction to: BMC Pulm Med (2020) 20:48**

**https://doi.org/10.1186/s12890-020-1081-6**

Following publication of the original article [1], the authors flagged that the name provided for the 4th author had been misspelled; the 4th author was (incorrectly) spelled as ‘Eyob Alemayehu Gebreyohanns’.

Please note that the correct spelling is ‘Eyob Alemayehu Gebreyohannes’.

The spelling has since been corrected in the original article and, furthermore, is provided in the author list of this correction.

The authors apologize for any inconvenience caused.
