# Supplementary material for: Barriers to and competency with the use of metered dose inhaler and its impact on disease control among adult asthmatic patients in Ethiopia
Source: BMC Pulm Med. 2020 Feb 21;20:48. doi: 10.1186/s12890-020-1081-6 (PMC7035747; doi:10.1186/s12890-020-1081-6)
Supplement: Supplementary file 1 — Additional file 1. Barrier and competency assessment questionnaire (BCA-Q). [file 12890_2020_1081_MOESM1_ESM.docx]

| No | Barriers | Yes | | no |
| --- | --- | --- | --- | --- |
| Cognition | | | | |
| 1 | The belief of the patient that their asthma is not serious to take the medication | |  |  |
| 2 | Fears of addiction or dependence to their medication | |  |  |
| 3 | The perception that medication should be used in response to symptoms, and not on a regular basis | |  |  |
| 4 | The belief of decreasing effectiveness of the medication over time | |  |  |
| 5 | Barrier was inadequate or limited knowledge about their medication: | |  |  |
| 6 | The fear of adverse effects of medication associated with use of an inhalers | |  |  |
| **7** | Inadequate or limited knowledge whether they were taking the right medication. | |  |  |
| 8 | Inadequate or limited knowledge whether they were using the appropriate technique | |  |  |
| 9 | Inadequate or limited knowledge whether the medication they were taking was compatible with medications taken for other conditions | |  |  |
| Motivation and preferences | | | | |
| 10 | Forgetfulness |  | |  |
| 11 | Lack of motivation |  | |  |
| 12 | preference for other non-pharmacological approach |  | |  |
| 13 | preference for restriction of daily physical activity instead of taking medication |  | |  |
| Practical implementation | | | | |
| 14 | having to brush their teeth after its use |  | |  |
| 15 | uncomfortable for the use of chambers |  | |  |
| 16 | Having trouble to take the medication more than once a day |  | |  |
| 17 | The cost of medication |  | |  |
| Family and physician related barriers | | | | |
| 18 | Disagreements between parents |  | |  |
| 19 | Unclear language and instruction |  | |  |
| 20 | Uninformed about the disease or diagnosis |  | |  |
| 21 | the severity of the disease remained unclear to them |  | |  |
| 22 | physicians centered the asthma management strategy solely on prescribed medications |  | |  |
| Health care related barriers | | | | |
| 23 | the lack of a structured follow-up plan |  | |  |
| 24 | lack of specialists treating asthma |  | |  |
| 25 | contradictory messages provided by different health care professionals |  | |  |
